# Is Exercise during Pregnancy a Risk for Gestational Age and Preterm Delivery? Systematic Review and Meta-Analysis

**DOI:** 10.3390/jcm12154915

**Published:** 2023-07-26

**Authors:** Rubén Barakat, Dingfeng Zhang, Miguel Sánchez-Polán, Cristina Silva-José, Javier Gil-Ares, Evelia Franco

**Affiliations:** 1 AFIPE Research Group, Faculty of PA and Sport Sciences-INEF, Universidad Politécnica de Madrid, 28040 Madrid, Spain; barakatruben@gmail.com (R.B.); zhangdingfeng123@gmail.com (D.Z.); miguelsanpol@gmail.com (M.S.-P.); cristina.silva.jose@upm.es (C.S.-J.); 2 Department of Education, Research and Evaluation Methods, Faculty of Social and Human Sciences, Universidad Pontificia de Comillas, 28049 Madrid, Spain; efalvarez@comillas.edu

**Keywords:** pregnancy, exercise, gestational age, preterm delivery

## Abstract

Traditionally, one of the primary concerns regarding exercise during pregnancy has been the potential of reducing gestational age and increasing the likelihood of preterm delivery. The aim of this study was to perform a systematic review about the effects of physical activity (PA) during pregnancy on gestational age and preterm delivery. A systematic review and two meta-analyses were performed (Registration No. CRD42022370770). Data sources from online databases were searched up to November 2022. The review exclusively included studies involving pregnant populations and interventions consisting of PA implemented during pregnancy. The primary outcomes analysed were gestational age, measured in weeks, and the occurrence of preterm deliveries. A total of 57 studies were analysed through two independent meta-analyses for the first one, no association was found between moderate exercise during pregnancy and gestational age (Z = 0.45, *p* = 0.65, ES = 0.08, 95% CI = −0.06−0.04, I2 = 42%, P heterogeneity = 0.001), showing the exercise group had a higher gestational age. In addition, no differences were found between groups in terms of number of preterm deliveries (RR = 0.96, (95% CI = 0.77–1.21, Z = 0.33, *p* = 0.74; ES = 0.07; I2 = 31%, P heterogeneity = 0.05)). The findings of this study indicate that there is no association between exercise during pregnancy and reduced gestational age or increased risk of preterm delivery in healthy pregnancies.

## 1. Introduction

The intricate process of pregnancy and childbirth plays a crucial role in shaping the long-term health outcomes for both the mother and the child. Spanning over several months, it involves substantial modifications in nearly all of a woman’s bodily systems to support the growth and development of the foetus. Therefore, it is imperative to ensure the optimal functioning of all maternal physiological, mental, and emotional mechanisms that facilitate foetal growth and development. Any complications arising within these domains of health may contribute to pathologies and complications that adversely affect the well-being of both the mother and the newborn [1,2].

The numerous demands imposed by pregnancy transform the process of gestation and childbirth into a formidable challenge that women must navigate throughout the forty weeks of gestational age. Their goal is to avert pathologies and adverse outcomes, such as preterm delivery, which can give rise to significant associated complications [3,4].

Premature birth refers to the condition where a baby is born before the completion of a full-term pregnancy, which lasts approximately 40 weeks. Preterm birth, on the other hand, occurs when delivery takes place prior to the 37th week of pregnancy. Premature babies often encounter significant health challenges, particularly when born at earlier stages of gestation. The nature and severity of these problems can vary, with a higher risk of associated health complications observed as the gestational age decreases [5]. In this intricate scenario, it is crucial for pregnant women to prioritize the well-being of all aspects of their body, encompassing not only physiological factors but also mental and emotional aspects [6].

In light of scientific evidence, it is well-established that unhealthy lifestyles have negative implications for pregnancy outcomes. Engaging in an unhealthy lifestyle during pregnancy heightens the risk of chronic diseases [7]. Moreover, the escalating epidemic of obesity and sedentary habits significantly impacts pregnancy and childbirth, with enduring adverse consequences [8]. The scientific literature highlights the substantial benefits derived from various forms of physical activity (PA) in terms of pregnancy outcomes and overall health and well-being [9,10]. Nevertheless, the impact of PA during pregnancy on gestational age and preterm delivery has not been extensively investigated, leaving significant scientific gaps [11].

Achieving an optimal gestational age and preventing a preterm delivery are two crucial factors that significantly impact the well-being of both the mother and child during the pre-, peri-, and postnatal periods. Traditionally and historically, physical exercise during pregnancy has been inaccurately perceived as a challenging factor for both gestational age and preterm birth.

PA has become an integral component for diverse populations, including pregnant women. Many studies have confirmed the benefits of PA on different maternal, foetal, and newborn outcomes. Nevertheless, the impact of various types of PA on gestational age and preterm delivery has been inadequately examined through systematic reviews, resulting in a significant knowledge gap in this scientific domain. Through a rigorous analysis of the recent literature, it is evident that only a few studies with high reliability, such as systematic reviews with meta-analyses, have been conducted. Thus, studies with a rigorous scientific methodology and guaranteed reliability are essential to generate new evidence on this issue.

In this sense, in the recent scientific literature, only one systematic review study [12] has specifically investigated the effects of gestational PA on preterm delivery. The objective of this systematic review and meta-analysis is to examine the current scientific evidence concerning the effects of PA during pregnancy on gestational age and the occurrence of preterm delivery.

## 2. Materials and Methods

This study was developed following the Preferred Reporting Items for Systematic reviews (PRISMA) guidelines [13] and registered with the International Prospective Register of Systematics reviews (PROSPERO, registration No. CRD42022370770). Population, Intervention, Comparison, Outcomes and Study design framework (PICOS) was used to analyse the searching sources included in this research [14].

### 2.1. Population

Pregnant women without any obstetrical relative (e.g., gestational hypertension, malnutrition, or moderate cardiovascular disease) or absolute (e.g., premature labour, preeclampsia, or incompetent cervix) contraindications, participating in a PA programme during pregnancy were chosen.

### 2.2. Intervention

The intervention characteristics analysed were: (a) weekly frequency of PA sessions; (b) intensity: all studies included had a moderate intensity of load, using 55–65% of the maximum maternal heart rate or the perception of effort (range 12–14 of the Borg Scale); (c) duration of the PA program; (d) type of PA (e.g., yoga, Pilates, aerobic, strength, or pelvic floor training); (e) supervision or not of the PA program; (f) time duration of the sessions, as shown in Table 1.

### 2.3. Comparison

Women who engaged in an exercise or PA program during pregnancy were compared with those who did not participate in such a program. Intervention characteristics were retrieved and compared as shown in Table 1.

### 2.4. Outcomes

The gestational age (measured in weeks) and the preterm deliveries were the target outcome.

### 2.5. Study Design and Selection Process

The search for this study was done between September and November 2022, at Universidad Politécnica de Madrid (INEF). EBSCO (including Academic Search Premier, Education Resources Information Center, MEDLINE, SPORTDiscus, and OpenDissertations databases), Clinicaltrials.gov, Web of Science, Scopus, Cochrane Database of Systematic Reviews, and Physiotherapy Evidence Database (PEDro) were searched. More precisely, articles written in English or Spanish and published between 2010 and 2022 were searched.

The search terms were:

- English: physical activity OR exercise OR physical exercise OR fitness OR strength training OR physical intervention OR cointervention AND pregnancy OR pregnant OR maternal OR antenatal AND randomized clinical trial OR RCT OR non-randomized clinical trial AND gestational age OR preterm birth OR preterm delivery.
- Spanish: actividad física OR ejercicio OR ejercicio físico OR fitness OR entrenamiento de fuerza OR intervención física OR co-intervención AND embarazo OR embarazada OR maternal OR prenatal AND ensayo clínico aleatorizado OR ensayo clínico no aleatorizado AND edad gestacional OR nacimiento a pretérmino OR parto pretérmino.

**Figure 1:**
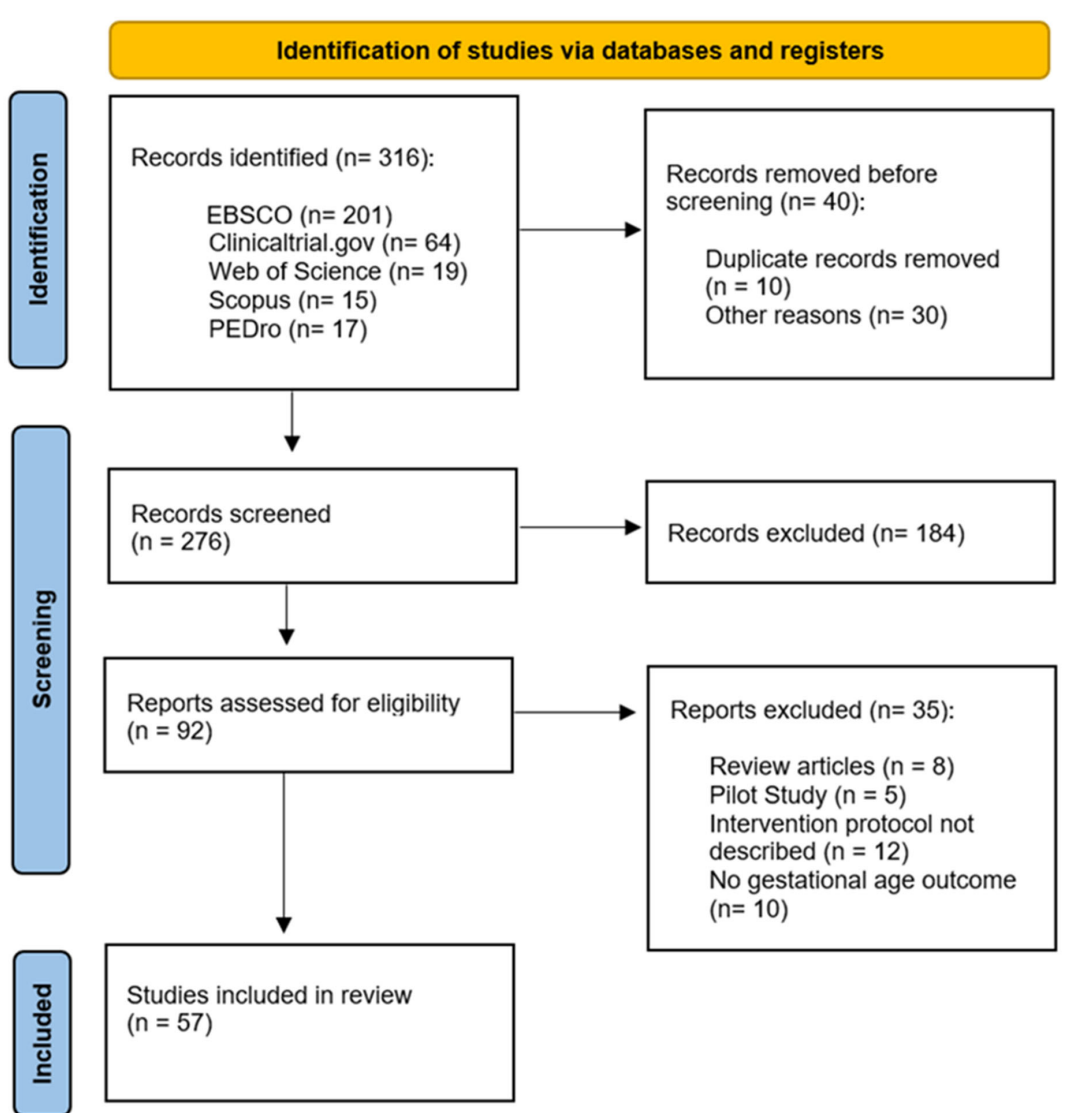
Flow diagram of the analysed articles.

Eligible articles for our review included studies that had a quantifiable PA or exercise intervention (excluding the articles with only advice to have an active pregnancy or those having a measurable PA questionnaire but without an exercise intervention), with gestational age or preterm delivery as outcomes, and with the characteristics of the PA or exercise program provided. This process is detailed in Figure 1.

The primary outcomes were gestational age and preterm birth. Studies reporting either of them were included in the review. Firstly, two reviewers (M.S.-P. and D.Z.) screened independently the studies retrieved in the search achieving a complete consensus in the decision about the eligible studies. In a second stage, two reviewers (M.S.-P. and C.S.) performed the data extraction from the included studies. In case of doubt at that stage, they consulted with the rest of the authors until an agreement on the appropriate manner to report the information from the study was reached. In cases where both gestational age and preterm delivery were reported in the same study, both measures were separately included in the meta-analysis. Additionally, to check the effects of each intervention on maternal health, other outcomes were examined (but not included in the meta-analyses) as secondary outcomes, such as physiologic, sociodemographic, and delivery outcomes. From each chosen study, we extracted the author(s), publication year, country in which the study was developed, type of study design, number of participants, characteristics of the intervention program, and variables analysed (primary and secondary) (Table 1).

### 2.6. Statistical Analysis, Quality of Evidence Assessment, and Risk of Bias

As mentioned earlier, two separate meta-analyses were performed. For the first meta-analysis including the studies reporting gestational age as a continuous variable, the overall confidence interval (CI) was calculated using the standardized mean difference [15]. For the second meta-analysis, the dependent variable was the ratio of preterm deliveries in each study, and it was expressed as a categorical variable (yes/no). In that case, the number of events present in each study group and its relative risk (RR) were recorded, and the total sum of the RR was calculated using a random-effects model [16]. In both analyses, each study had a relative weight assignment corresponding to its sample size number, which contributed to the entire analysis, establishing the compensated average. The I2 statistic was used to quantify the heterogeneity present in the results due to the different interventions and designs of each article, indicating the variability of the effect of each intervention, and whether it was random or not. The following considerations were used: low heterogeneity = 25%; moderate heterogeneity = 50%; and high heterogeneity = 75% [17]. Ferreira-González et al. [18] demonstrated that in the case of a high heterogeneity, one solution could be to divide the studies into subgroups performed with different characteristics explaining that variability. However, for our article with limited results, we understood that presenting all articles in each analysis would provide a better approach for the study.

For the assessment of the quality of evidence for the main outcome and each study, the Grading of Recommendations Assessment, Development and Evaluation (GRADE) framework was used, including studies rated as having a moderate or high quality [19]. To determine the potential risk of bias (with these sources: selection, performance, attrition, detection, and reporting bias), the Cochrane Handbook was followed [20]. Randomised clinical trials’ evidence initially started with a “low” risk of bias (due to the theoretical study design and intervention), compared to nonrandomised interventions, and both increased or decreased its risk of bias in function of having any “high” or “low” score across bias sources. Both the quality of the study and the risk of bias analyses were performed by three of the reviewers (M.S.-P., C.S., and E.F.).

**Table 1:** Characteristics of the studies included in the review.

Author	Year	Country	N	EG	CG	Intervention Features							Main Variables Analysed	Secondary Variables Analysed
						Freq	Intensity	PT	Type	Superv. Class	Duration	Adh.		
Babbar [21]	2016	US	46	23	23	3	Mod	8 w	Yoga	Yes	60 min	-	Gestational age, type of delivery, birth weight	Gestational weight gain
Bacchi [22]	2018	Argentina	111	49	62	3	Low–mod	26 w	Aquatic activities	Yes	55–60 min	80%	Gestational weight gain, gestational age	Birth weight
Backhausen [23]	2017	Denmark	516	258	258	2	Low	12 w	Aerobic	No	70 min	-	Low back pain, birth weight	Gestational age, type of delivery
Barakat [24]	2011	Spain	80	40	40	3	Low–mod	28 w	Aerobic and light strength training	Yes	35–45 min		Maternal health status	Gestational age, type of delivery
Barakat [25]	2012 a	Spain	290	138	152	3	Mod	28 w	Aerobic exercise	Yes	40–45 min	-	Type of delivery	Gestational age and birth weight
Barakat [26]	2012 b	Spain	83	40	43	3	Low–mod	28–33 w	Land aerobic and aquatic activity	Yes	35–45 min	-	Gestational weight gain and gestational diabetes	Gestational age, type of delivery, birth weight
Barakat [27]	2013	Spain	510	255	255	3	Mod	28 w	Aerobic, strength, and flexibility exercise	Yes	50–55 min	-	Gestational diabetes, gestational age	Gestational weight gain and birth weight
Barakat [28]	2014 a	Spain	290	138	152	3	60–75% Max HR	28–31 w	Aerobic exercise	Yes	55–60 min	80%	Gestational age	Gestational diabetes, gestational weight gain
Barakat [29]	2014 b	Spain	200	107	93	3	Mod	26–31 w	Aerobic exercise	Yes	55–60 min	80%	Gestational age, gestational weight gain, type of delivery, gestational diabetes	Birth weight, head circumference
Barakat [30]	2016	Spain	765	382	383	3	Mod	28 w	Aerobic, strength, and flexibility exercise	Yes	50–55 min	80%	Hypertension	Type of delivery, gestational age, birth weight
Barakat [31]	2018 a	Spain	429	227	202	3	Mod	28 w	Aerobic exercise	Yes	55–60 min	80%	Duration of labour, gestational age	Type of delivery, use of epidural, birth weight
Barakat [32]	2018 b	Spain	65	33	32	3	Mod	28 w	Aerobic, pelvic floor strength, and flexibility exercises	Yes	55–60 min	-	Placenta’s weight	Gestational age, type of delivery, birth weight
Barakat [33]	2019	Spain	456	234	222	3	Mod	28 w	Aerobic exercise	Yes	50–55 min	-	Gestational weight gain	Gestational age, type of delivery, birth weight
Bhartia [34]	2019	India	78	38	40	1 2	Mod	12 w	Yoga	Yes No	50 min	-	Maternal stress, type of delivery, birth weight	Gestational age
Bjøntegaard [35]	2021	Norway	281	164	117	1–2	Mod–high	12 w	Endurance, strength training and balance exercises	Yes No	60 min 45 min	-	Type of delivery, birth weight, gestational age	PA of children at age of seven
Brik [36]	2019	Spain	85	42	43	3	Light–mod	29 w	Aerobic, strength, and pelvic floor exercises	Yes	60 min	70%	Gestational weight gain	Type of delivery, birth weight, gestational age
Carrascosa [37]	2021	Spain	286	145	141	3–5	55–65% Max HR	20 w	Water aerobic exercise	Yes	45 min	-	Use of epidural analgesia during labour	Type of delivery, time of active labour, episiotomy, gestational age
Clark [38]	2018	USA	36	14	22	3	Mod	20 w	Aerobic	Yes	60 min	-	Gestational weight gain, gestational age	Type of delivery, birth weight
Cordero [39]	2012	Spain	55	25	30	3	50–55% Max HR	28–33 w	Aerobic and strength training	Yes	50 min	80%	Gestational weight gain, gestational diabetes,	Gestational age
Cordero [40]	2015	Spain	342	122	220	3	Mod	24 w	Aerobics in a gym hall and aquatic activity	Yes	50–60 min	80%	Gestational diabetes, gestational age	Gestational weight gain, type of delivery, birth weight
Da Silva [41]	2017	Brazil	639	213	426	3	Mod	16+ w	Aerobic, strength and stretching training	Yes	60 min	70%	Gestational age, preterm birth, and pre-eclampsia	Gestational weight gain, birth weight
Daly [42]	2017	Ireland	88	44	44	3	Mod	8 w	Aerobic pelvic floor exercises	Yes	50–60 min	-	Maternal fasting plasma glucose, gestational age	Type of delivery and birth weight
de Barros [43]	2010	Brazil	64	32	32	1–2	Mod	12 w	Resistance exercise	Yes No	30 min	-	Gestational diabetes, gestational age	-
Dias [44]	2011	Norway	42	21	21	1	Low	16 w	Pelvic floor muscle training	Yes	30 min	75%	Type of delivery, birth weight, gestational age	Pelvic floor muscle strength
Ellingsen [45]	2020	Norway	279	164	115	1–2	Mod	12 w	Aerobic activity and strength exercises	Yes No	60 min 45 min	-	Neurodevelopmental in 7-year-old children	Gestational age, birth weight, type of delivery
Fernández-Buhigas [46]	2020	Spain	92	41	51	3	50–60% Max HR	23–27 w	Aerobic, strength, coordination and balance, pelvic floor exercises	Yes	60 min	80%	Blood pressure, gestational weight gain	Gestational age
Ghodsi [47]	2014	Iran	80	40	40	3	50–60% Max HR	12–18 w	Stationary cycling	No	15 min	-	Gestational age, type of delivery, perineal tear	-
Guelfi [48]	2016	Australia	169	84	85	3	65–75% Max HR	14 w	Home-based stationary cycling program	Yes	60 min	-	Gestational diabetes	Gestational age, type of delivery, birth weight
Haakstad [49]	2011	Norway	105	52	53	2–1	Mod	12 w	Aerobic dance and strength training	Yes No	60 min 30 min	-	Birth weight	Gestational age, type of delivery
Halse [50]	2015	Australia	40	20	20	3	55–65% Max HR	6 w	Stationary cycling	No	45 min	-	Maternal attitude and intentions of exercise	Gestational age, gestational weight gain
Hellenes [51]	2015	Norway	336	188	148	1–3	Mod	16 w	Aerobic activity	Yes No	30+ min	-	Cognitive, language and motor domains of children	Gestational age, birth weight, and type of delivery
Kong [52]	2014	USA	37	18	19	5	Mod	20 w	Treadmill walking	No	30 min	-	Postpartum weight retention	Gestational weight gain, gestational age, birth weight
Leão [53]	2022	Brazil	424	141	283	3	Mod	16 w	Aerobic and strength training	Yes	60 min	70%	Childhood neurodevelopment	Gestational age
McDonald [54]	2021	USA	192	131	61	3	Mod	24+ w	Aerobic and resistance training	Yes	50 min	-	Infant Morphometry	Gestational age
McMillan [55]	2019	USA	60	33	27	3	Mod	20 w	Aerobic activity	Yes	50 min		Infant neuromotor skills	Gestational weight gain, gestational age
Murtezani [56]	2014	Repub. of Kosovo	63	30	33	3	Mod	20 w	Aerobic and strength exercises	Yes	40–45 min	-	Birth weight, gestational age	-
Nascimento [57]	2011	Brazil	82	40	42	1–5	Low–mod	22 w	Aerobic exercise, walking	Yes No	40 min	-	Gestational weight gain, gestational age	Birth weight
Nobles [58]	2015	USA	251	124	127	5	Mod	12 w	Aerobic activity	No	30 min	-	Gestational diabetes	Birth weight, gestational age
Pais [59]	2021	India	132	66	66	7	Low	14–18 w	Yoga	No	45 min	-	Preeclampsia and gestational diabetes	Gestational age, analgesia, duration of labour, type of delivery, birth weight
Perales [60]	2015	Spain	63	38	25	3	55–60% Max HR	26–31 w	Aerobic dance, pelvic floor muscle training	Yes	55–60 min	-	Foetal and maternal heart rate	Gestational weight gain, gestational age, birth weight, type of delivery
Perales [61]	2016	Spain	166	83	83	3	55–60% Max HR	28–31 w	Aerobic and strength exercises	Yes	55–60 min	-	Duration of labour, gestational age, type of delivery, birth weight	-
Perales [62]	2020	Spain	1348	668	660	3	Light–mod	30 w	Aerobic, pelvic floor exercises	Yes	50–55 min	-	Gestational weight gain, hypertension and gestational diabetes	Type of delivery, birth weight, gestational age, preterm delivery
Raper [63]	2021	USA	125	58	67	3	Mod	22 w	Aerobic	Yes	50 min	80%	Gestational diabetes, type of delivery and birth weight	Gestational age
Renault [64]	2014	Denmark	283	142	141	7	Low	22–26 w	11,000 daily steps	No	60+ min	-	Gestational weight gain, miscarriage	Gestational age
Ruchat [65]	2012	Canada	71	26	45	2–3	Mod	22 w	Walking	Yes No	25–40 min	-	Gestational weight gain, birth weight, gestational age	-
Ruiz [66]	2013	Spain	96	48	48	3	Light–mod	29–30 w	Aerobic and resistance exercises	Yes	50–55 min	-	Gestational weight gain, gestational age	Birth weight, type of delivery
Sagedal [67]	2017	Norway	591	296	295	2	Mod	24 w	Strength training and cardiovascular exercise	Yes	60 min	-	Gestational weight gain, birth weight	Gestational age
Sanda [68]	2018	Norway	589	295	294	2–3	Mod	22 w	Aerobic exercises	Yes No	60 min 30 min	-	Gestational age, duration of labour, type of delivery	-
Seneviratne [69]	2015	New Zealand	75	38	37	3–5	Mod	16 w	Stationary cycling program	No	15–30 min	-	Birth weight, type of delivery	Gestational weight gain, gestational age
Silva-Jose [70]	2022	Spain	157	78	79	3	Mod	28–31 w	Aerobic exercise	Yes	55–60 min	80%	Gestational weight gain, gestational age	Type of delivery, birth weight
Stafne [71]	2012	Norway	761	396	365	1–3	Mod	12 w	Aerobics, strength, pelvic floor exercises	Yes No	60 min 45 min	-	Urinary and anal incontinence	Type of delivery, birth weight, gestational age
Taniguchi [72]	2016	Japan	118	60	58	3	Mod	6+ w	Walk briskly	Yes	30 min	80%	Type of delivery, birth weight	Gestational age
Tomic [73]	2013	Croatia	334	166	168	3	60–75% Max HR	28–30 w	Aerobic exercise	Yes	50 min	80%	Macrosomia, birth weight, type of delivery, gestational weight gain	Gestational age
Uria-Minguito [74]	2022	Spain	203	102	101	3	65–70% Max HR	28–31 w	Aerobic exercise	Yes	50–60 min	-	Gestational diabetes, gestational age	Gestational weight gain, type of delivery, birth weight
Ussher [75]	2015	UK	789	394	395	3–4	Low	6 w	Exercise on treadmill	Yes	20 min	-	Continuous smoking abstinence	Type of delivery, birth weight
Wang [76]	2017	China	300	150	150	3	55–65% Max HR	18 w	Stationary cycling program	Yes	60 min	75%	Gestational diabetes	Birth weight, macrosomia, gestational age
Yekefallah [77]	2021	Iran	70	35	35	2	Low–mod	11 w	Yoga	Yes	75 min	-	Episiotomy, type of delivery	Birth weight, gestational age, duration of labour

Author last name (Ref). Year: year of study. Country: country where the article has been developed (usually in the method part). Type: type of article; if it is a randomized clinical (or controlled) trial, put RCT; if not, please specify the item. N: total number of women analysed. Those of the GI and those of the CG have to coincide. GI: number of women analysed in the intervention group. GC: number of women analysed in the control group. Freq: weekly frequency of exercise sessions (3 days a week, 2, etc.). Intens: type of intensity, e.g., moderate, high… Tp: program time; if the program has lasted 10 weeks, or if it has started in week 12 and ends in week 28, put it as 16 weeks long. Type: type of exercise performed, e.g., aerobic, muscle strengthening, etc. Superv. classes: whether or not there was supervision. Duration: minutes of each session. Adh.: adherence of the participants to the intervention (%); it is how and how many sessions women have attended. Main variables analysed: lists all the main variables of the study. It is usually in the method section in “outcomes”, and they appear as “main outcomes”. If main variables do not appear, they are the first. One can find it in several places. Secondary variables: the same as before but secondary.

## 3. Results

In total, 276 articles were retrieved in the first stage of the search, 184 of which were excluded because they did not meet the inclusion criteria (Figure 1). Then, 35 articles were excluded since they were a narrative review (*n* = 8), a pilot study (*n* = 5), they did not describe the intervention protocol (*n* = 12), or they did not provide information regarding gestational age (*n* = 10). Finally, 57 studies were included in the analysis.

**Figure 2:**
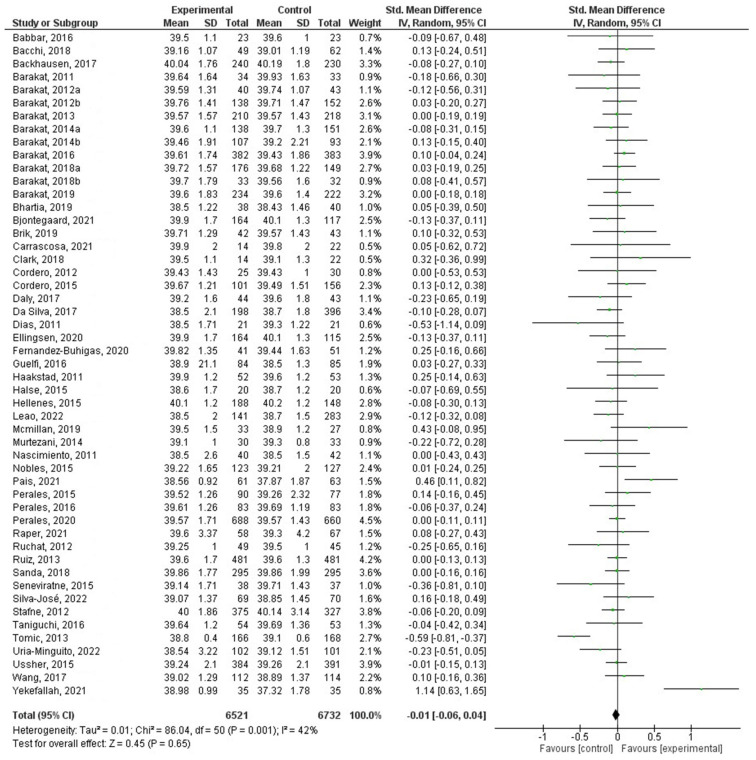
Effect of PA during pregnancy on gestational age [21,22,23,24,25,26,27,28,29,30,31,32,33,34,35,36,37,38,39,40,41,42,44,45,46,48,49,50,51,53,55,56,57,58,59,60,61,62,63,65,66,68,69,70,71,72,73,74,75,76,77].

**Figure 3:**
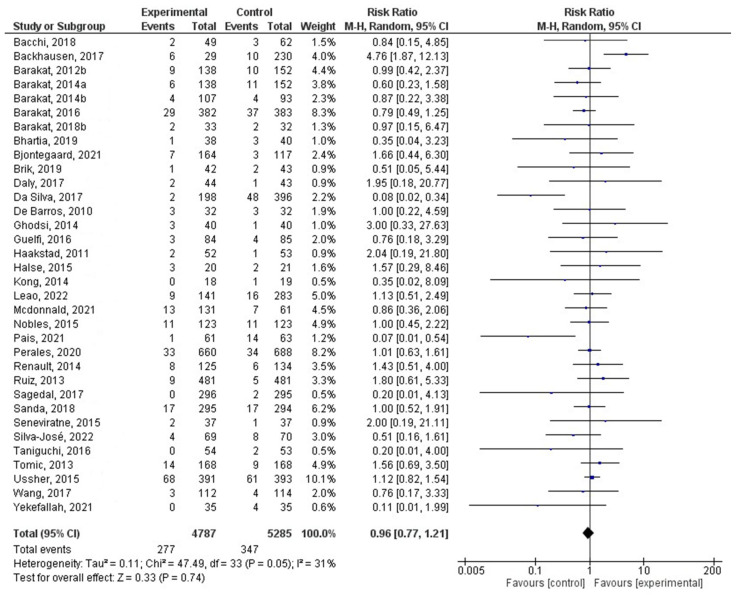
Effect of PA during pregnancy on the ratio of preterm deliveries [22,23,26,28,29,30,32,34,35,36,41,42,43,47,48,49,50,52,53,54,58,59,62,64,66,67,68,69,70,72,73,75,76,77].

For the first meta-analysis, all the selected studies were included that reported gestational age (Figure 2). On the other hand, 34 of them were represented in the second meta-analysis (Figure 3). In this group, studies reporting whether the women had had a preterm delivery were included.

Regarding the type of intervention reported in the included studies (as shown in Table 1), most of them described PA sessions conducted by professionals in the field. The intervention consisted of aerobic exercise, strength exercises, or aquatic activities among others. The sessions in the studies included in the review were designed for moderate intensity and performed with a frequency of one to seven days per week, with a time duration between 15 and 75 min. The duration of each intervention oscillated between 2 and 24 weeks.

In terms of the quality of evidence assessed using the GRADE (Grading of Recommendations Assessment, Development, and Evaluation) approach, a total of 52 randomized controlled trials (RCTs) were analysed for the assessment of gestational age. The findings of these trials resulted in a classification of a “moderate” certainty and “critical” importance. Similarly, for the analysis of preterm delivery, 34 RCTs were evaluated, which provided a classification of a “high” certainty and “critical” importance.

### 3.1. Effect of PA on Gestational Age

Fifty-two different studies were included in this analysis, comparing gestational age between women in the experimental and control groups. The results revealed there was no significant association between exercise practice during pregnancy and gestational age (Z = 0.45, *p* = 0.65; ES = 0.08; 95% CI = −0.01 [−0.06–0.04]; and the values for heterogeneity analysis were Chi^2^_50_ = 86.04 (*p* = 0.001), I2 = 42%). Figure 2 shows the forest plot corresponding to the present meta-analysis.

### 3.2. Effect of PA on the Risk of Preterm Delivery

Thirty-four studies were included in this analysis comparing the difference in the ratio of preterm deliveries between the experimental and control groups. The results revealed that there was no association between PA practice during pregnancy and preterm delivery risk. Specifically, the total compensated RR was 0.96 (95% CI = 0.77–1.21, Z = 0.33, *p* = 0.74: ES = 0.07), and the values for heterogeneity analysis were Chi^2^_33_ = 47.49 (*p* = 0.05), I2 = 31%). These outcomes indicate that women who exercised during pregnancy did not present a significantly greater probability of experiencing preterm delivery. Figure 3 shows the forest plot corresponding to the present meta-analysis.

### 3.3. Risk of Bias Assessment

**Figure 4:**
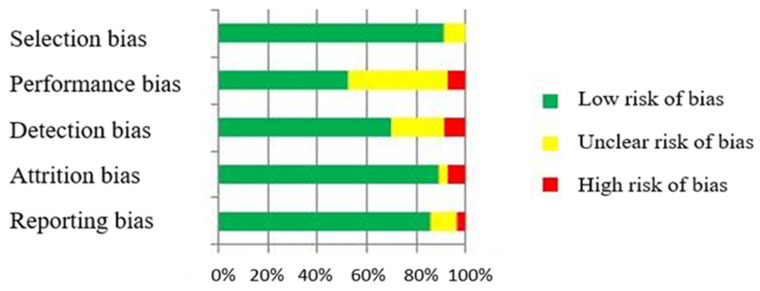
Risk of bias.

Overall, the risk of bias of each article was rated as low, unclear, or as a high potential risk (Figure 4). Reviewing the sources of bias, the articles showed mostly a low risk of bias on selection, detection, and attrition bias. Nearly a half of the studies (*n* = 27) presented an unclear performance risk. In this type of research (controlled trials), blinding participants is practically impossible. On the other hand, a high (*n* = 5) or unclear (*n* = 12) selection bias was reported for some studies due to the difficulty to access each protocol. Despite the high percentage of unclear risk of bias findings in the performance source, we followed Cochrane’s tool considering the difficulty in these types of studies when it comes to blinding participants [20]. Despite the high risk of bias scored in the reporting source, the outcomes of interest of each article (even the inaccessibility of their protocols) could not be related to gestational age or preterm delivery. The summary of the risk of bias assessment per study is included as Supplementary Materials (File S1).

## 4. Discussion

The objective of the present systematic review and meta-analysis was to investigate the impact of PA during pregnancy on gestational age and the occurrence of preterm delivery. By assessing the potential risks to maternal and foetal well-being associated with an integral component of a pregnant woman’s daily life, namely PA, we aimed to enhance our understanding of its influence.

Our findings indicated that there were no adverse effects observed on both gestational age and the occurrence of preterm delivery. In fact, the exercise groups demonstrated a lengthened gestational age compared to the control groups, which is consistent with findings from other studies [11]. This is particularly significant because traditionally, a major concern surrounding exercise during pregnancy has been the potential for a decreased gestational age and increased risk of preterm delivery. However, our results support the recommendation of moderate PA throughout pregnancy for pregnant women without obstetric contraindications, aligning with a substantial body of literature [78,79,80].

From an epidemiological standpoint, when examining the underlying causes of the beneficial impact of physical exercise on gestational age, we encountered ample evidence to support a causal association between unhealthy lifestyles—such as excessive maternal weight gain, obesity, and smoking—and the occurrence of preterm delivery [81,82,83].

These findings lead us to propose the concept of a comprehensive preventive effect of PA throughout pregnancy, mitigating the risk factors associated with reduced gestational age and preterm birth. This notion is supported by other studies demonstrating the potential of PA to positively impact not only physiological aspects but also mental and emotional aspects, and the overall quality of life in pregnant women [84,85].

The results of this study further validate the current recommendations of engaging in 150 min of moderate PA per week throughout pregnancy for women without obstetric contraindications [79].

While no significant limitations of the evidence or the review processes used in the current study were identified, there are two minor limitations worthy of note. The first one refers to the diversity of interventions involving PA across the included studies; the absence of standardized protocols hampers the potential of meta-analyses to their fullest extent. On the other hand, the review included both women who exercised before and women who started exercising during pregnancy. However, we believe that this variability is inherent in the nature of the intervention itself.

## 5. Conclusions

PA during pregnancy does not contribute to adverse outcomes in terms of gestational age or increase the risk of preterm delivery among healthy pregnant women.

## Data Availability

Not applicable.

## Supplementary Materials

The following supporting information can be downloaded at: https://www.mdpi.com/article/10.3390/jcm12154915/s1, File S1. Risk of bias; File S2. PRISMA 2020 Checklist.
