# Supplementary material for: Is Exercise during Pregnancy a Risk for Gestational Age and Preterm Delivery? Systematic Review and Meta-Analysis
Source: J Clin Med. 2023 Jul 26;12(15):4915. doi: 10.3390/jcm12154915 (PMC10419377; doi:10.3390/jcm12154915)
Supplement: Supplementary file 1 [file jcm-12-04915-s001.zip › jcm-2489477-File S1.pdf]

	Babbar et al.	Bacchi et al.	Backhausen et al.	Barakat 2010 et añ	Barakat 2011 et al.	Barakat 2012 a et al.	Barakat 2012 b et al.	Barakat 2013 et al.	Barakat 2014 a et al.	Barakat 2014b et al.	Barakat 2016 et al.	Barakat 2018a et al.	Barakat 2018b et al.	Barakat 2018c et al.	Bhartia et al.
Selection bias	+	+	+	-	+	+	+	+	+	+	+	+	+	+	?
Performance bias	+	+	+	-	?	?	?	+	-	?	?	+	+	+	?
Detection bias	+	+	+	+	?	-	+	+	-	-	-	+	+	+	+
Attrition bias	+	+	+	+	+	+	+	+	+	+	+	+	+	+	+
Reporting bias	+	+	+	-	?	+	+	+	+	?	+	+	+	+	+
	?	+	+	+	?	?	+	+	?	?	?	+	+	+	-

	Bjontegaard et al.	Brik et al.	Carrascosa et al.	Clark et al.	Cordero et al.	Cordero et al.	Da Silva et al.	Daly et al.	Dias et al.	Ellingsen et al.	Fernandez-Buhigas et al.	Garnæs et al.	Guelfi et al.	Haakstad et al.	Halse et al.
Selection bias	+	+	+	+	?	?	+	+	+	+	+	+	+	+	+
Performance bias	+	+	+	?	?	?	+	?	+	+	?	+	+	+	?
Detection bias	+	+	+	+	?	?	+	?	?	+	?	-	+	+	+
Attrition bias	+	+	+	+	+	+	+	+	+	+	+	+	+	+	+
Reporting bias	+	+	+	+	+	+	+	+	?	+	+	+	+	+	+
Other	-	+	+	-	+	+	+	?	+	-	+	+	+	+	+

	Hellenes et al.	Leao et al.	Murtezani et al.	McDonald et al.	McDonald et al.	McMillan et al.	Nascimiento et al.	Nobles et al.	Pais et al.	Perales et al.	Perales et al.	Perales et al.	Raper et al.	Ruchat et al.	Ruiz et al.
Selection bias	+	+	+	+	?	+	+	+	+	+	+	+	+	+	+
Performance bias	?	+	+	?	?	?	+	?	+	+	?	-	?	?	-
Detection bias	+	+	+	+	+	+	?	+	+	+	?	+	+	+	-
Attrition bias	+	+	-	+	+	+	+	+	+	+	+	+	+	-	+
Reporting bias	+	+	+	+	+	+	+	+	+	+	+	+	+	+	+
OTHER	-	+	?	+	+	?	+	+	+	+	+	+	+	?	+

	Sanda et al.	Seneviratne et al.	Silva-José et al.	Sobhgol et al.	Stafne et al.	Taniguchi et al.	Tomic et al.	Uria-Minguito et al.	Ussher et al.	Nobles et al	Vinter et al	Wang et al.	Yekefallah et al.
	+	+	+	+	+	+	+	+	+	+	+	+	+
Selection bias	?	+	+	+	+	?	+	+	+	?	+	-	+
Performance bias	+	+	+	?	+	?	+	+	+	+	+	-	?
Detection bias	+	+	+	+	+	+	+	+	+	+	+	+	-
Attrition bias	+	?	+	+	+	+	-	+	-	+	+	?	+
Reporting bias	?	+	+	+	+	?	-	+	?	+	?	?	-
